# Hematologic malignancies masquerading as rheumatologic diseases: A case series and review

**DOI:** 10.1097/MD.0000000000042251

**Published:** 2025-04-25

**Authors:** Julia Clark, Makshada Kowlessur, Elysha VanderVeer, Kamran Shojania, Sheila Au, Hyein Kim, Shannon Wong, Kun Huang

**Affiliations:** a Department of Medicine, University of British Columbia, Vancouver, British Columbia, Canada; b Arthritis Research Canada, Vancouver, British Columbia, Canada; c British Columbia Cancer Agency, Surrey, British Columbia, Canada; d Division of Rheumatology, University of British Columbia, Vancouver, British Columbia, Canada; e Department of Dermatology and Skin Science, University of British Columbia, Vancouver, British Columbia, Canada; f Division of Nephrology, University of British Columbia, Vancouver, British Columbia, Canada.

**Keywords:** hematologic malignancy, multiple myeloma, myelodysplastic syndrome, rheumatologic syndromes

## Abstract

**Rationale:**

Hematologic malignancies can mimic rheumatologic diseases, presenting a significant diagnostic challenge due to overlapping clinical features. This study highlights 5 cases of hematologic malignancies presenting as rheumatologic disorders and discusses the diagnostic complexities involved.

**Patient concerns:**

The patients, aged 64 to 78, presented with diverse rheumatologic symptoms including polyarthritis, vasculitis, Raynaud phenomenon, and systemic symptoms such as weight loss, fatigue, and night sweats. Initial workups suggested rheumatologic diagnoses, leading to delays in recognizing the underlying malignancies.

**Diagnoses:**

The diagnostic journey involved extensive laboratory testing, imaging, and, in all cases, bone marrow biopsies, which ultimately revealed hematologic malignancies: angioimmunoblastic T-cell lymphoma (AITL), extranodal marginal zone lymphoma, myelodysplastic syndrome (MDS), and multiple myeloma. Misleading initial findings, such as autoimmune serologies and transient responses to immunosuppressive therapy, complicated the diagnostic process.

**Interventions:**

Ultimately, the patients included in this case series benefited from hematological malignancy-specific therapies. Delayed diagnosis impacted the treatment course and outcomes.

**Outcomes:**

Outcomes varied: 2 patients achieved symptom control with targeted therapy, while others experienced complications such as infections or disease progression, ultimately leading to mortality in some cases. Patient frustrations underscored the psychologic toll of diagnostic delays.

**Lessons:**

Hematologic malignancies can present as atypical or refractory rheumatologic diseases, emphasizing the need for vigilance in patients with unusual clinical courses. Early consideration of malignancy in differential diagnoses, especially with atypical serologic or histopathologic findings, is critical to improving outcomes.

## 1. Introduction

Paraneoplastic phenomena occur in approximately 10% of patients with solid tumors and are mediated by malignancies via mechanisms other than direct tumor invasion. For example, dermatomyositis may arise in association with ovarian, lung, gastric, or nasopharyngeal cancer. Hypertrophic osteoarthropathy is frequently associated with lung or gastrointestinal cancers. When solid tumors are suspected, advanced imaging techniques, including computed tomography (CT), positron emission tomography (PET), and magnetic resonance imaging (MRI), facilitate the timely detection of tumor types.

In contrast, liquid tumors pose greater diagnostic challenges. Autopsy studies have revealed that critical diagnoses are often missed in complex hematologic cases, with a significant proportion of these missed diagnoses potentially altering the management or prognosis.^[1]^ Additionally, misdiagnosis rates for hematologic neoplasms range from 15% to 40% when samples are reviewed by non-specialist pathologists, with lymphoma subtype misclassification being most common, followed by incorrect classification of malignant lesions as benign or vice versa.^[2,3]^ This high diagnostic discordance highlights the inherent difficulty of accurately diagnosing hematologic malignancies. When such malignancies mimic rheumatologic disorders, the diagnostic challenge for care providers intensifies.

Hematologic malignancies manifesting as rheumatologic diseases represent a diagnostic conundrum, often presenting with diverse and overlapping features. Conditions such as polyarthritis, migratory arthritis, and giant cell arteritis (GCA) have been reported in multiple myeloma, angioimmunoblastic T-cell lymphoma (AITL), and acute myeloid leukemia.^[4]^ MDS can also present with systemic vasculitis, connective tissue disease (CTD), inflammatory arthritis, or neutrophilic dermatosis.^[5]^ In one report, chronic myelomonocytic leukemia (CMML) mimicked systemic lupus erythematosus (SLE), presenting with polymyalgia rheumatica, myositis, and cutaneous vasculitis, further complicated by overlapping features such as thrombocytopenia and positive antinuclear antibodies (ANA).^[6]^ Notably, hematologic malignancies may initially respond well to corticosteroids and immunosuppressive agents, further obscuring the distinction from primary rheumatic conditions.^[4]^

We present a series of 5 patients with hematologic malignancies masquerading as rheumatologic diseases and review the relevant literature. These cases underscore the importance of considering hematologic malignancies in patients with atypical rheumatologic presentations, particularly when the clinical course is unusual or refractory to immunosuppressive therapy.

## 2. Methods

We conducted a retrospective review of 5 patients who presented to a general rheumatology clinic between January 2021 and April 2024 with rheumatologic findings and were subsequently diagnosed with a hematologic malignancy. Detailed data on the clinical presentation, laboratory findings, imaging results, treatments, and both initial and revised diagnoses were collected. The study was approved by the University of British Columbia Research Ethics Board (Study number H24-01665), and written consent for case reporting and imaging was obtained from each patient and/or their guardian.

For the literature review, we searched MEDLINE, EMBASE, and Web of Science using the following keywords: hematologic malignancies, rheumatic diseases, paraneoplastic rheumatic diseases, hematologic malignancies mimic rheumatic diseases. Truncation symbols were used where appropriate to capture variations in terminology. We included case reports, case series, reviews, and original research articles published between the years 1974 and 2025 in English language. Conference abstracts were included if they contained significant clinical information. Duplicate articles retrieved from multiple databases were eliminated before the screening process. Studies deemed not relevant to the topic were excluded after a thorough review of their abstracts and full texts.

## 3. Results

Demographic data, clinical findings, autoimmune serologies, initial rheumatologic diagnoses, and eventual hematologic malignancies are summarized in Table 1.

**Table 1:** Summary of clinical and demographic characteristics of patients.

Patient Sex	1 Female	2 Female	3 Male	4 Male	5 Male
Age at symptom onset (yr)	74	66	77	77	56
Age current or at death (yr)	76	67	81	79	64
Race	Caucasian	Caucasian	Caucasian	Caucasian	Caucasian
Presenting symptoms and signs	Polyarthralgia, fever, weakness, hemoptysis, ITP	LCV rash, constitutional symptoms, progressive anemia and renal dysfunction	Polyarthritis, weakness, constitutional symptoms, progressive macrocytic anemia, and bullous neutrophilic dermatosis	RPC, polyarthritis, constitutional symptoms, cranial GCA like symptoms, progressive macrocytic anemia	Raynaud phenomenon, digital ischemia, sclerodactyly
Autoimmune serologies	ANA 17 SSA > 240	Type II cryoglobulin	Anti-PL-7 medium positive	All negative	All negative
Abnormal hematological parameters	ITP	Normocytic anemia	Normocytic anemia	Macrocytic anemia	Normocytic anemia
Initial rheumatological diagnosis	Undifferentiated CTD, with vasculitis features	Cryoglobulinemic vasculitis	PMR then autoinflammatory disorder NOS	Giant cell arteritis	Systemic sclerosis
Rheumatology treatments	IVIg, glucocorticoid, IV cyclophsophamide	Prednisone	Prednisone, methotrexate, azathioprine, mycophenolate mofetil, cyclosporine, tofacitinib and sarilumab	Prednisone	Adalat and topical sildenafil
Final hematological diagnosis	Angioimmunoblastic T-cell lymphoma	MALT lymphoma	Myelodysplastic syndrome	Myelodysplastic syndrome	Multiple myeloma
Time between initial and final diagnosis	16 mo	3 mo	26 mo	12 mo	3 mo
Hematological treatments	Brentuximab vedotin, cyclophosphamide, doxorubicin, and prednisone. Subsequently declined any further therapy	Bendamustine and rituximab	None	None	Bortezomib, lenalidomide, dexamethasone
Outcome	Alive	Alive	Deceased	Alive	Alive

### 3.1. Patient 1: Polyarthralgia, hemoptysis, and immune thrombocytopenia (ITP) with positive SSA antibody

A 74-year-old Caucasian female with a remote history of breast cancer presented to the emergency department in July 2022 with acute hemoptysis, on a background of 1 month of polyarthritis, subjective lower extremity weakness, petechial rash to her lower legs, and intermittent fever and chills. Initial laboratory tests showed severe thrombocytopenia (platelets < 5 × 10^9^/L) and normocytic anemia (Hemoglobin nadir: 69 g/L); of note, she had normal platelet and hemoglobin levels 2 weeks prior to admission. Renal function, liver enzymes, bilirubin, and lactate dehydrogenase were normal. An autoimmune work-up showed an elevated C-reactive protein (CRP) of 37.1 mg/L, an ANA ratio of 17.0, and a positive SSA antibody (>240). Antineutrophil cytoplasmic antibodies (ANCA) were negative. Epstein–Barr virus IgG and IgM antibodies were reactive. Serum protein electrophoresis (SPEP) showed a polyclonal increase in gamma-globulins. Flow cytometry of the peripheral blood was nondiagnostic for lymphoproliferative disorders, and the remainder of the autoimmune and viral serologies were unremarkable. Imaging included chest CT, which demonstrated extensive bilateral airspace disease and enlarged mediastinal lymph nodes (up to 23 mm) thought to be inflammatory or infectious. Abdominal CT revealed no abnormalities or splenomegaly.

She was diagnosed with ITP secondary to systemic vasculitis or undefined CTD. Initial treatment included intravenous (IV) methylprednisolone (1 g daily for 3 days), IV immunoglobulin (2 g/kg), tranexamic acid 10mg/kg, and 16 platelet transfusions. Despite these treatments, her platelet counts remained critically low (<5 × 10^9^/L) but her arthralgias, rash, and constitutional symptoms had resolved. Cyclophosphamide induction therapy (750 mg/m^2^) was initiated, resulting in normalization of her platelet count 3 weeks after induction therapy. She completed 3 additional rounds of monthly IV cyclophosphamide infusions that were dose-reduced and eventually discontinued due to intolerable mucositis. She subsequently declined any further immunosuppressive therapy and rheumatology follow-up.

One year later in August 2023, the patient re-presented to the emergency department with progressive night sweats, chills, and shortness of breath. Repeat chest imaging revealed worsening lymphadenopathy and new findings of intra-abdominal lymphadenopathy and splenomegaly. Excisional lymph node biopsy confirmed AITL, and bone marrow biopsy demonstrated moderate involvement. Despite initiating standard chemotherapy with brentuximab vedotin (1.8 mg/kg), IV cyclophosphamide (750 mg/m^2^), IV doxorubicin (50 mg/m^2^), and oral prednisone (45 mg/m^2^) (CHP-BV), her condition deteriorated after 2 cycles. Shared decision making between her lymphoma provider and patient was to transition to best supportive care and received no further chemotherapy.

### 3.2. Patient 2: Progressive anemia, acute renal failure, and cryoglobulinemic vasculitis

A 66-year-old previously healthy female presented with a 1-month history of palpable purpura and lower extremity edema in July 2023. Skin biopsy of her right lower extremity confirmed leukocytoclastic vasculitis. Cryoglobulins (Type II), consisting of monoclonal IgM kappa and polyclonal IgG, were detected. Other than decreased C4 (0.04 g/L), her remaining bloodwork was unremarkable, including CBC, renal function, electrolytes, CRP, and ANA.

She was diagnosed with cryoglobulinemic vasculitis which spontaneously resolved without any therapy. By October 2023, she developed daily drenching night sweats, and experienced severe fatigue. A drop in her serum hemoglobin level from 120 to 71 g/L and rise in her creatinine level from 64 to 221 μmol/L were noted on bloodwork. A bone marrow biopsy revealed < 5% involvement of low-grade B-cell lymphoma, which could not be further classified with no evidence of plasma cell neoplasm or amyloid deposition. A whole body CT showed no lymphadenopathy or splenomegaly. PET revealed no 18F-fluorodeoxyglucose (FDG) avid malignancy.

The modest bone marrow involvement in low-grade B-cell lymphoma was not in keeping with the degree of her debilitating constitutional symptoms, rapid progressive anemia, and renal dysfunction. This prompted a diagnostic renal biopsy in January 2024, which revealed severe acute tubular injury and interstitial edema. However, there was no evidence of paraprotein-related kidney disease or cryoglobulinemic glomerulonephritis. Further pathological review at a specialized cancer center confirmed multifocal lymphoid aggregates in nodular and interstitial distribution with features consistent with primary renal involvement of extranodal marginal zone lymphoma of mucosa-associated lymphoid tissue (MALT lymphoma).

She was initially treated with prednisone 50 mg daily, followed by 6 cycles of monthly bendamustine (90 mg/m^2^ on days 1 and 2) and rituximab (375 mg/m^2^ on day 1 or 2) with complete resolution of skin manifestations and constitutional symptoms, and improvement in renal function, anemia, and neuropathy.

### 3.3. Patient 3: polyarthritis, generalized weakness, constitutional symptoms, progressive macrocytic anemia, and bullous neutrophilic dermatosis

A previously healthy 77-year-old male from a remote community was first evaluated by rheumatology in May 2021 for a 10-month history of proximal muscle weakness and atrophy, polyarthritis affecting the shoulders, hips, knees, and small joints of the hands, as well as progressive weight loss. He also had developed an erythematous, nontender, nonpruritic papular rash over his fingers, chest, shoulder, and legs. Initial bloodwork revealed very mild normocytic anemia (hemoglobin, 122 g/L) but significantly elevated CRP (190 mg/L). The left knee synovial fluid aspirate showed a total nucleated cell counts of 2611 × 10^6^/L which was minimally inflammatory. Creatine kinase, ANA, ENA, rheumatoid factor (RF), anti-cyclic citrullinated peptide, ANCA, cryoglobulin, and complements were all negative or within normal limits. SPEP showed polyclonal gammopathy with no monoclonal bands. Myositis specific antibody was moderately positive for PL-7, but electromyography (EMG) testing was negative for myopathic changes and MRI of his thighs showed no edema. A high-resolution CT (HRCT) of the chest showed no interstitial lung disease and pulmonary function testing was normal. No solid organ malignancy was detected on colonoscopy or CT of the chest, abdomen, or pelvis. Ultrasonography of the carotid, subclavian, and axillary arteries was negative for large vessel vasculitis. Cytokine testing revealed elevated IL-6 at 17.5 pg/mL (N = 0.2–9.4) and IL-8 at 32.1 pg/mL (N = 1.5–16.2).

**Figure 1.**
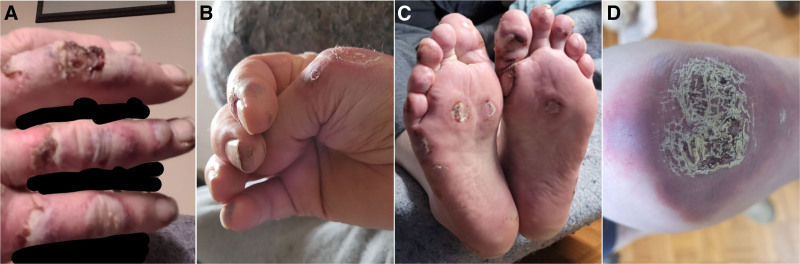
Photos of Patient 3 with rash on the hands, feet and right elbow. (A) Necrotic bullous lesions on the dorsum of the fingers. Interdigital spaces have been blacked out to mask the patient face. (B) Violaceous black thrombotic papules on the fingertips. (C) Necrotic papules and bullous lesion on the toes and sole of the feet. (D) A large violaceous scaly plaque over the right elbow.

Throughout the year of 2022, his rash evolved from erythematous based papules to non-follicular based inflammatory pustules and bullae involving his digits, arms, ears, and nose (Fig. 1). The patient had violaceous black thrombotic papules on the fingertips and ear pinnae, digital ulcers, and widespread livedo reticularis. A skin biopsy of the left forearm lesion confirmed neutrophilic dermatosis.

At the initial symptom onset, he was diagnosed with polymyalgia rheumatica (PMR) by his general practitioner. After review by the rheumatologist, the diagnosis was revised to an autoinflammatory disorder not otherwise specified. His arthritis and rash were responsive to high dose prednisone (~40 mg/d) but recurred when prednisone was tapered to 15 mg/d or lower. From 2021 to 2023, he was treated with several disease modifying agents, including methotrexate (up to 25 mg sc weekly), azathioprine (150 mg daily), mycophenolate mofetil (MMF) (1.5 g twice daily), cyclosporine (2.5 mg/kg daily), and targeted immunotherapy including tofacitinib (5 mg twice daily) and sarilumab (200 mg sc every 2 weeks); all with varying dosages of prednisone. Initially, all therapies provided partial symptomatic improvement, but never complete resolution of his arthritis, weakness or rash.

While treating his inflammatory symptoms, he had worsening anemia (hemoglobin nadir 68 g/L) with mild macrocytosis (MCV 105 fl), that eventually became transfusion-dependent. VEXAS (vacuoles, E1 enzyme, X-linked, autoinflammatory, somatic) syndrome testing for variants in the UBA1 gene yielded negative results. Given the atypical presentation and lack of response to all immunosuppressive agents, a bone marrow biopsy was performed in February 2023 which revealed an isochromosome of the long arm of chromosome 17 (17q) and abnormal localization of immature precursors, both of which supported the diagnosis of MDS.

Unfortunately, due to underlying MDS and immunosuppressive therapies including prolonged glucocorticoid exposure for over 2 years, he had several infectious complications including COVID-19 associated pneumonia, community-acquired pneumonia, and later disseminated *Mycobacterium chelonae* bacteremia. He failed to clear the mycobacterial infection despite several months of antibiotics; as a result, all immunosuppressives were held and chemotherapy for his MDS was never initiated. He eventually passed away 9 months later due to complications from his ongoing infection and progression of his hematologic malignancy.

### 3.4. Patient 4: recurrent relapsing polychondritis, migratory polyarthritis, giant cell arteritis like symptoms, night sweats, and macrocytic anemia

A 78-year-old male with a history of stage III chronic renal disease secondary to biopsy-proven membranous nephropathy, previously remotely treated with 2 courses of rituximab, presented with 1 year of recurrent and self-remitting polychondritis affecting the nose and both ears. At symptoms onset, he also had a concurrent episode of diplopia, jaw pain, and temporal scalp tenderness associated with CRP elevation of 208 mg/L. A temporal artery biopsy was negative, but GCA was diagnosed clinically and was treated empirically with prednisone (60 mg daily), tapering over of approximately 9 months that resolved his symptoms completely.

In February 2023, 3 months after discontinuing prednisone, he began to develop recurrent left ear chondritis, migratory inflammatory arthritis, and frequent night sweats. His CRP had improved but was still elevated at 70.5 mg/L. This was when he was first seen by a rheumatologist and was restarted on a prednisone taper starting at 30 mg daily, which again led to prompt symptom remission that was maintained with low dose prednisone 5 mg daily.

He had progressive macrocytic anemia for 4 years with a hemoglobin nadir of 76 g/L and an MCV of 104 fl. With epoetin alfa injections, his hemoglobin level improved to 120 g/L. Renal function was stable at a baseline eGFR of 30 mL/min. ANCA, ANA, dsDNA, RF and cryoglobulin levels were negative or within normal limits. SPEP demonstrated a normal pattern. VEXAS syndrome was suspected but NextGen sequencing of peripheral blood showed no UBA1 mutation.

In May 2023, he underwent a bone marrow biopsy for his anemia; findings were suggestive of MDS with dysplastic features in approximately 10% to 15% of megakaryocytes with no dysplasia in erythrocytes or granulocytes. Some immature granulocytes with cytoplasmic vacuolization were identified but no increase in blasts was observed. The myeloid panel and cytogenetic assessment did not reveal any abnormalities.

The patient’s diagnosis was revised to low-risk MDS with secondary relapsing polychondritis and arthritis. He is maintained on low dose prednisone 5 mg with complete control of the inflammatory symptoms. He continues to be followed up by hematology and remains on surveillance for low risk MDS.

### 3.5. Patient 5: long standing Raynaud phenomenon, finger digital ischemia, sclerodactyly and dermal sclerosis

**Figure 2.**
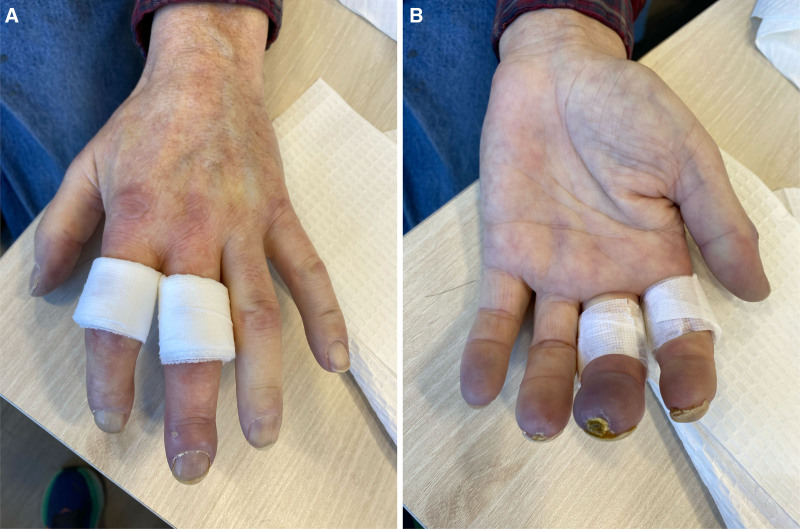
Photos of the left hand in Patient 5. Puffy fingers and skin thickening in areas distal to the MCPs. Dressings were applied to sites of skin biopsy. Cyanosis was visible in multiple fingers with ischemic lesions in middle and ring finger.

A 64-year-old male with an 8-year history of Raynaud phenomenon presented to rheumatology in March 2024 with progressive ischemic changes in the fingers, associated with skin thickening (Fig. 2). He had constitutional symptoms with a weight loss of 40 lb over 6 to 12 months, night sweats, subjective fevers, and rigors. He denied any symptoms of esophageal reflux and there was no interstitial lung disease on imaging. There were no small waxy papules elsewhere that would suggest scleromyxoedema. Investigations showed normocytic anemia with a hemoglobin level of 96 g/L and an elevated CRP level at 55 mg/L. Renal function and calcium levels were normal. ANA, ENA, Double-stranded DNA, RF, anti-CCP, cryoglobulins, and extended scleroderma autoantibody panel were all negative. Skin biopsy of the dorsum of the hands confirmed dermal sclerosis, which was consistent with sclerodermoid changes. Nailfold video capillaroscopy revealed a late scleroderma pattern with low density and significant capillary dropouts.

The provisional diagnosis at this time was seronegative scleroderma. He was administered nifedipine (60 mg daily) and topical sildenafil 5% for severe Raynaud symptoms and digital ischemia, only results in partial symptomatic relief.

A work-up for plasma cell dyscrasia was ordered for his normocytic anemia; the SPEP showed a dominant (29 g/L) IgG kappa band in the mid-gamma zone, and the UPEP also showed a kappa light chain monoclonal protein. He had elevated kappa free light chains at 1267.6 mg/L (N = 3.3–19.4) with a free kappa to lambda light chain ratio of 91.86 (N = 0.26–1.65). There were no osteolytic lesions or bone-based plasmacytoma on CT skeletal survey. A bone marrow biopsy showed clonal plasma cells with an aberrant phenotype on flow cytometry and 28% involvement of abnormal clonal plasma cells on immunohistochemistry, confirming plasma cell myeloma. Congo red staining was negative for amyloidosis.

His diagnosis was revised to be multiple myeloma with secondary skin sclerosis, Raynaud phenomenon, and digital ischemia. He was initiated on chemotherapy with bortezomib (1.5 mg/m^2^ weekly), lenalidomide (10 mg daily), and dexamethasone (40 mg weekly), leading to significant improvement in his Raynaud and digital ischemia symptoms.

In all 5 cases, patients expressed frustration over the delay between their initial symptom concerns and the eventual diagnosis of the underlying hematologic malignancies.

## 4. Literature review

Various hematologic malignancies have been reported to present with rheumatologic manifestations. A total of 211 articles from MEDLINE, 34 from EMBASE, and 85 from Web of Science were extracted. We excluded duplicate articles, non-English publications, and studies deemed not relevant to the topic. Furthermore, case reports were excluded if they described either the coexistence of hematological malignancies and rheumatological diseases or malignancies arising as complications of systemic autoimmune diseases. Only cases where hematological malignancies mimicked rheumatological conditions were considered. Thirty-four articles including case reports, reviews, and abstracts were analyzed in detail for the literature review. A summary of the rheumatologic syndromes associated with hematologic malignancies is presented in Table 2.^[4–37]^

**Table 2:** Literature review of hematological malignancies masquerading as rheumatologic diseases.

Hematological diagnosis	Presenting rheumatological symptoms	References
Lymphoma	Polyarthritis	^[4,7–20]^
	Systemic vasculitis	
	Leukocytoclastic vasculitis	
	Polymyositis/dermatomyositis	
	RS3PE	
	SLE-like syndrome	
	Raynaud phenomenon	
Myelodysplastic syndrome	Polyarthritis	^[5–7,13,18,21–26]^
	Systemic vasculitis	
	Leukocytoclastic vasculitis	
	PMR	
	Relapsing polychondritis	
	SLE-like syndrome	
	RS3PE	
	Neutrophilic dermatosis	
	Pyoderma gangrenosum	
	Sweet syndrome	
Multiple myeloma	Polyarthritis	^[4,19,20,27–33]^
	Systemic vasculitis	
	Leukocytoclastic vasculitis	
	Scleroderma	
	Polymyositis/dermatomyositis	
Leukemia	Polyarthritis	^[4,8,10,11,13,19,20,25,26,34–37]^
	Systemic vasculitis	
	Scleroderma	
	R3SPE	

RS3PE = remitting seronegative symmetrical synovitis with pitting edema.

## 5. Discussion

Hematologic malignancies have long been associated with autoimmune pathology, manifesting in diverse ways such as asymptomatic autoantibodies, inflammatory symptomatology, or established rheumatologic diagnoses. These malignancies can mimic rheumatologic diseases through various mechanisms, often occurring concurrently in the same patient. For instance, Patient 2 demonstrated direct B-cell renal invasion alongside paraneoplastic cryoglobulinemic cutaneous vasculitis. Similarly, the case of Patient 1 is with AITL, a recognized mimic of SLE, highlights the role of monoclonal expansion of follicular T helper cells and dysregulated antibody responses in this overlap.^[38,39]^

In MDS, the imbalance between regulatory T (Treg) cells and inflammatory T helper type 17 (Th17) cells in early low-risk disease underlies the high prevalence of autoimmune phenomena.^[21,40]^ VEXAS syndrome exemplifies the genomic connection between MDS and autoinflammatory symptoms via somatic mutations in UBA1, disrupting E1 enzyme activity. Plasma cell dyscrasias, including multiple myeloma and monoclonal gammopathies, can present with symmetric polyarthritis owing to mechanisms such as monoclonal light chain deposition in synovial tissue^[30]^ or IL-6 overexpression.^[32,41]^ B-cell lymphomas with pulmonary lymphoid granulomatosis may present as granulomatosis with polyangiitis, further complicating the diagnosis.^[42]^ Consequently, the multifaceted mechanisms through which hematologic malignancies mimic rheumatic diseases pose a significant diagnostic challenge owing to their broad spectrum of clinical presentations.

The 5 cases presented here reflect this formidable challenge. Immunologic laboratory abnormalities, such as ANA positivity, direct antiglobulin tests, lupus anticoagulant, and complement reductions (C3, C4), frequently bias clinicians toward rheumatologic diagnoses.^[24,43]^ Moreover, hematologic abnormalities may be absent (as in Patient 2) or subtle (as in Patient 3) at initial presentation. For example, autoimmune cytopenias secondary to hematologic malignancy in Patient 1 responded to glucocorticoids, cytotoxic agents, and IVIg, which reinforced an anchoring bias toward a rheumatologic etiology. Anemia, particularly in older patients, is often attributed to chronic diseases such as renal failure (Patients 2 and 4) or inflammation (Patients 3 and 5). Additionally, systemic glucocorticoids are toxic to lymphocytes and plasma cells and are part of the treatment of many hematologic diseases. Therefore, it is not surprising that patients with hematologic malignancies presenting with rheumatologic disease also display a favorable response to glucocorticoids.

Through case analysis and literature review, we identified several clinical clues indicative of hematologic malignancy rather than a primary rheumatologic condition:

1. Predominant constitutional symptoms such as rapid weight loss and drenching night sweats.
2. Severe, disabling symptoms with incomplete response to glucocorticoids and DMARDs.
3. Worsening cytopenias and increased transfusion requirements.
4. New, progressive lymphadenopathy, splenomegaly, and/or paraproteinemia.
5. Multiorgan involvement and atypical autoimmune presentations inconsistent with a single rheumatologic diagnosis.
6. Male patients who presented with relapsing polychondritis, GCA, polyarteritis nodosa, neutrophilic dermatoses, and macrocytic anemia.
7. Detection of multiple autoantibodies in the same patient, with clinical presentations that do not align with antibody profiles or span distinct autoimmune diseases.

In summary, we emphasize the importance of recognizing these clinical clues and thoroughly investigating their potential for hematologic malignancies. This may necessitate extensive imaging and multiple biopsies, with a specialized pathology review of atypical biopsy sites strongly recommended. Effective diagnosis and management hinge on the close collaboration between rheumatologists and hematologists.

## Author contributions

**Conceptualization:** Julia Clark, Kun Huang.

**Data curation:** Julia Clark, Kun Huang.

**Investigation:** Julia Clark, Elysha VanderVeer, Kamran Shojania, Sheila Au, Hyein Kim, Shannon Wong, Kun Huang.

**Methodology:** Julia Clark, Makshada Kowlessur, Kun Huang.

**Project administration:** Julia Clark, Makshada Kowlessur, Kun Huang.

**Supervision:** Kun Huang.

**Validation:** Elysha VanderVeer, Kamran Shojania, Sheila Au, Hyein Kim, Shannon Wong, Kun Huang.

**Writing – original draft:** Julia Clark, Kun Huang.

**Writing – review & editing:** Julia Clark, Makshada Kowlessur, Elysha VanderVeer, Kamran Shojania, Sheila Au, Hyein Kim, Shannon Wong, Kun Huang.
